# Mental health: health worker narratives in the context of COVID-19 in Brazil

**DOI:** 10.47626/1679-4435-2023-1130

**Published:** 2023-11-24

**Authors:** Daniele Mometti-Braz, Rosana Teresa Onocko-Campos

**Affiliations:** 1 Programa de Pós-Graduação em Saúde Coletiva, Faculdade de Ciências Médicas, Universidade Estadual de Campinas (UNICAMP), Campinas, SP, Brazil; 2 Departamento de Saúde Coletiva, Faculdade de Ciências Médicas, UNICAMP, Campinas, SP, Brazil

**Keywords:** health personnel, mental health, COVID-19, pessoal da saúde, saúde mental, COVID-19

## Abstract

**Introduction:**

During the pandemic, “staying at home” was not an option for many people, especially health workers, who were on the front lines in the fight against COVID-19 and whose mental health was threatened.

**Objectives:**

This study highlights the psychological repercussions of the pandemic and the coping strategies used by health workers in an effort to develop mental health resources.

**Methods:**

This qualitative exploratory study investigated the narratives of 14 health workers from an online focus group between August and September 2020, transcribing the meetings and performing thematic content analysis.

**Results:**

The analysis resulted in 3 categories of experience: 1) the professional as a patient, especially the fear of suffering or dying based on daily experience; 2) the fine line between providing care and being a vector of transmission, which identified worries about infecting family members and work overload; and 3) coping strategies and mental health on an individual level, such as routines, care, faith, and learning lessons, as well as on a collective level, such as prevention measures and public policies.

**Conclusions:**

Studies such as this can contribute to a framework of knowledge about coping strategies to maintain mental health among health workers. They also provide an opportunity to listen, which produces meaning, empathy, and recognition for these professionals.

## Abstract (pt)

**Introdução:**

Em tempos de pandemia, para muitos “o ficar em casa” não foi bem uma opção, tendo como exemplo trabalhadores da área da saúde que estiveram na linha de frente do combate à COVID-19, com a própria saúde mental posta à prova.

**Objetivos:**

Dar visibilidade ao construto das repercussões psicológicas e às estratégias de enfrentamento, contribuindo para o reconhecimento da vivência desses profissionais e para a construção de futuros saberes.

**Métodos:**

Pesquisa exploratória, de abordagem qualitativa, possibilitando empatizar, via narrativas, a vivência de 14 trabalhadores da saúde, por meio de grupo focal on-line, realizado entre agosto e setembro de 2020, com encontros transcritos e análise temática de conteúdo.

**Resultados:**

A análise das narrativas resultou em três categorias sobre a vivência desses trabalhadores: 1) O profissional enquanto paciente, destacando-se o medo de sofrer ou morrer baseado no que vivenciavam no dia a dia; 2) O exercício da função e a linha tênue entre o cuidado e a transmissão, identificando-se preocupações com o contágio de familiares e a sobrecarga de trabalho; e 3) Estratégias de enfrentamento e saúde mental, sendo apontadas questões de enfrentamento individual, como as rotinas, os cuidados, a fé, as aprendizagens, e de enfrentamento coletivo, as práticas preventivas e políticas públicas.

**Conclusões:**

Pesquisas como esta contribuem para um arcabouço de conhecimento quanto às estratégias de enfrentamento que favorecem a saúde mental do trabalhador, além de trazer oportunidade de escuta, produzindo sentido, empatia e reconhecimento a esses profissionais.

## INTRODUCTION

During the global pandemic of COVID-19, a previously unknown coronavirus, the world of work was deeply affected,^1^ especially health professionals on the frontlines of care. Any unfamiliar situation could be potentially damaging psychologically,^2^ requiring resilience, which, according to Walsh,^3^ goes beyond coping; it involves learning from previous crises, whether personal, family, social, or in the community. Thus, this study sought to give visibility to the experiences of health professionals, using their narratives to identify the psychological repercussions of the pandemic and the mental health coping strategies they used during this unusual point in history.

### HEALTH WORKERS

The category of health workers^4^ includes those who work in health services, health care, and health surveillance, whether in hospitals, basic health units, long-term care institutions, clinics, outpatient clinics, laboratories, or related institutions. This group includes the following professions: doctors, nurses, nutritionists, physical therapists, occupational therapists, biologists, biomedical scientists, pharmacists, dentists, speech therapists, psychologists, social workers, physical education professionals (who work in health units), veterinarians, and associated technicians and assistants. This group also includes support professionals, such as receptionists, security guards, cleaning workers, cooks, assistants, ambulance drivers, and funeral workers (who were in contact with potentially contaminated bodies).

There are approximately 3.5 million workers in Brazil’s Unified Health System (Sistema Único de Saúde) alone.^5^ According to the literature,^6^ health care professionals were 3 times more likely to become infected with COVID-19 than the general population. However, the problem went beyond physical issues. The professionals, faced with so much uncertainty about a new disease, constantly saw people entering and leaving services, trying (and regularly failing) to survive. Thus, mental health became a central issue for health workers during the pandemic.

### MENTAL HEALTH

According to World Health Organization data^7^ mental disorders, such as anxiety and depression, affected 264 million people worldwide prior to the pandemic. Hence, if mental health was already a challenge in “normal” times, imagine the repercussions during the pandemic!

A central characteristic of the pandemic was unpredictability, ie, uncertainty whether the established safety measures were sufficient to avoid infection and uncertainty whether one was actually infected or not, was serving as a vector of infection, would show symptoms, would be hospitalized, or would even survive. It goes without saying that such uncertainty could lead to anxiety and depression.

Beck’s cognitive model, among the most common ways to conceptualize and treat psychological disorders,^8^ contributes to the understanding that each person may interpret the pandemic situation differently. According to this model, “it is not the situation itself that determines what people feel, but how they interpret it”^9^ (p. 50). Thus, people’s thoughts influence their emotions and behavior.

Therefore, giving a voice to health care workers can increase our understanding of their thoughts and the strategies they employed in the chaos of the pandemic to deal with psychological suffering. This could lead to the development of mental health resources for these workers.

## METHODS

From a survey of a random and diverse sample of 56 Brazilians, this study will focus on the narratives of those who classified themselves as health professionals: a total of 14 participants of both sexes, including 3 men (2 doctors and 1 pharmacist) and 11 women (1 doctor, 1 resident, 5 nurses, 1 biomedical doctor, 2 psychologists, and 1 psychology intern). Ten of the workers were White and 4 were of mixed race; their ages ranged from 25 to 62 years, and they were from several cities in 2 states: São Luís (Maranhão) and Araras, Limeira, Rio Claro, and Campinas (São Paulo). The inclusion criteria were: health care professionals who worked during the pandemic and were available to participate in the study online.

The research was conducted between August and September 2020 through a focus group that met via videoconferencing (Google Meet), using participant observation and semi-structured interviews. The narratives were the result of 2 meetings lasting up to 1.5 hours each: the first was free conversation and exploratory questions about the pandemic experience, while the second was interpretive (hermeneutic), deepening and confirming the narratives from the first meeting.^10^

The hermeneutic narrative is a tool for exploring and searching for meaning in the experiences of the participating workers. According to Ricouer,^11^ narrative is a mediating operation between lived experience and discourse.

The narratives were investigated through content analysis,^12^ which, following Minayo,^13^ included 3 steps. The first step was pre-analysis of the collected material, including development of the textual corpus; attention was paid to the initial objectives, including preliminary reading of the collected material. The second stage was a deeper exploration of the materials, including an active search for the main expressions and their meanings, as well as the main actors in the narratives. Analytical categories were then selected. In the final stage, the results and their interpretation were prepared.

The respondents provided virtual informed consent prior to participation. The study was approved by the University of Campinas Human Research Ethics Committee (Comitê de Ética em Pesquisa em Seres Humanos da Universidade Estadual de Campinas - number 34443420.5.0000.5404).

## RESULTS AND DISCUSSION

Content analysis of the health workers’ statements about their experience during the pandemic led to the identification of 3 macro-categories, which are described below.

### THE PROFESSIONAL AS A PATIENT (EXPERIENCING THE DISEASE)

Constant exposure to the virus at work and, hence, suspecting personal infection caused as much suffering as actually being sick, as described by participant P5: “I took a test every 15 days, until one day it came back positive”. Either suspected or actual infection could severely restrict contact with family and friends, as in the case of one participating mother, whose son said “Mom, give me a kiss and a hug as a birthday present”. Her answer was “I can’t, son, you have to wait for the results”. She said that having to say this “made me sad” (P14).

To certain people, becoming infected “was just a matter of time” (P5). Some with more severe symptoms described more intense psychological suffering, since, due to their own experience with the cases they followed at work, they were already aware of what was to come: “I saw the intubation process in the ICU [intensive care unit]. I said: they’re going to intubate me, it’s horrible, I’m not going… I lost a lot of friends, my coworkers’ wives” (P6). “The tachycardia became more pronounced. I was very afraid of dying…I work in the health sector, I already knew what could happen” (P9).

From mild symptoms such as “just a headache” (P5) to “hospitalization, passing through the valley of the shadow of death” (P6), the disease was experienced in different intensities by the participants. Of the 14 participants, 50% had already been infected and 100% had already been isolated due to suspected infection. Those who had already been infected could not be sure they wouldn’t be infected again or that reinfection would not be even more severe.

### THE FINE LINE BETWEEN CARE AND TRANSMISSION

Although health professionals were publicly respected for their important role on the frontlines of the fight against COVID-19, this was soon overshadowed by the disturbing thought that their work conditions put them at higher risk and that, if infected, they might infect those they came in contact with, as expressed by P7: “The greatest concern was that the people who had contact with us would also become infected”. P5 stated “I was more worried about contaminating my family members than about being infected myself”. According to P2, “We worry about our parents, about our daughter. That’s what creates the most stress.”

Nurses who had recently given birth were worried about returning to work, since they would be exposed to the virus and could consequently expose their baby. They also feared their baby would be deprived of a mother if they became infected and had to be isolated, as expressed by P10: “and so if I get infected, who’s going to take care of him?” This vulnerable situation required intervention through public policy, which only occurred the following year, when Law 14,151 was approved^14^ on May 12, 2021, which guaranteed pay from the beginning of the maternity leave until 120 days after giving birth.

Unanimously, the health professionals were deprived of contact with people. Some considered this an act of love, which “beareth all things, believeth all things, hopeth all things, endureth all things”,^15^ including separation from one’s family. According to P3: “I didn’t go see my family to avoid contact” Some workers continued living with their family but did not get close to them: P6 reported “I couldn’t hug anyone anymore and no one could hug me”. Some suffered prejudice: “At the beginning of the pandemic, when the hospital became a reference center for COVID-19 patients, I felt a lot of prejudice. I even felt it from my family. I couldn’t visit some [of them]… [They gave me] looks… my friends, some jokingly, [but] others didn’t want to see me, because I worked at the hospital” (P11).

However, empathy and care among the professionals themselves increased: workers immediately returned from sick leave to alleviate their overloaded colleagues, as reported by P5: “[What has been even] more difficult, psychologically, was not my own illness, because I recovered, [but rather] knowing that I had to get back to work quickly, because it’s chaos! My colleagues are extremely overwhelmed and anxious. Another fighter out of combat.”

According to research from the University of São Paulo (Universidade de São Paulo),^16^ 6 out of 10 frontline health care professionals in the fight against COVID-19 were in mental distress. One cause was work overload due to the pandemic; another was fear of infection and organizational problems in health institutions, such as the lack of hospital beds and personal protective equipment.

Such exposure, in the midst of so many difficulties, can lead to mental illness.^17^ “Swallowing the tears” became synonymous with resilience in the harsh reality these professionals commonly faced in daily life: “I take care of ICU patients. I see colleagues leaving the room crying. Sometimes, we are caring for patients, all covered up [in protective equipment], and then we hear them asking: ‘Will I survive?’ ‘Do I have any chance?’ Sometimes, we have to swallow our tears and appear positive: ‘You’ll make it’” (P5).

### COPING AND MENTAL HEALTH

For frontline health workers, staying home was not an option. On the contrary, they had to look for strategies to deal with this unprecedented challenge. Their narratives indicated strategies they used to preserve their mental health while working during the pandemic.

### PREVENTION MEASURES AND PUBLIC POLICY

Although they have been the target of criticism, government initiatives, from the municipal to the federal level, did have an impact, such as recommendations for prevention measures, the use of masks, alcohol gel, social distancing (from others of unknown health status), and social isolation (from suspected or infected people).

Participants felt they needed greater support, including “more effective public policies” (P8), especially regarding isolation. According to P5: “It started too soon...and now that chaos is here...which is when we should close...they are opening. As a health care professional, I feel desperate, crazy!”

In an editorial, Maunder^18^ wrote that, although serious, the psychological impact of the 2002 severe acute respiratory syndrome outbreak on survivors was not a mental health catastrophe. This was mainly due to the fact that the virus was contained within a few weeks and that people did not transmit the disease when asymptomatic. Social restriction measures were mild and the scope of the outbreak was limited to certain countries, thus minimizing the scope of more serious mental health consequences.

Prevention measures, such as collective coping strategies, depended on individual care: “We take all possible precautions to avoid our own infection and retransmission within the hospital; all precautions are taken from the moment the patient enters” (P1), and “I think everyone ended up having a care ritual. I got home, took off my clothes, which were a little contaminated, you know, I went straight to the washing machine, [then] to the shower... I’m always wearing a mask” (P3).

These professionals needed help coping, and in some places specific programs were developed for health professionals, as mentioned by P8 (a psychologist): “We did more listening over the phone. A listening network was implemented in the hospital.” Listening by phone or videoconferencing was one strategy used to promote mental health. From May to September 2020, the Ministry of Health^19^ made the TelePSI project (a psychological teleconsultation service) available to health professionals.

Pan American Health Organization data^20^ show that the COVID-19 pandemic led a 25% higher prevalence of anxiety and depression worldwide. This new scenario must be addressed through appropriate strategies.

### MENTAL HEALTH CARE AND ROUTINES

The pandemic led to significant changes in daily life, not just affecting work routines, but revealing the need to develop work routines. “[N]ot just watching the news…I established routines so that my mind wouldn’t stay idle, thinking or worrying too much” (P13).

It was important for workers to avoid excess information or even misinformation, filtering what was heard and prioritizing what was discussed as a way of protecting the mind. Health professionals were constantly updated about the disease based on what they experienced at work, which was a reflection of what was happening around the world. “Take a little focus off bad news only” (P14). “It helped not to watch documentaries. The information I had was from my job, from the hospital” (P7). P2, who is married to P5 (both doctors), expressed it thus: “Our strategy is to try, when we are at home, not to talk about it. Most of the time we don’t succeed.”

The workers reported that hobbies and physical activity helped relieve stress. “Hobbies, anything that can bring some happiness and some balance” (P8). “Physical activity at home took away some of the anxiety. We see death every day, so it leads to anxiety, even depression. Friends of mine are suffering from depression in the hospital because they don’t want to work anymore. You have to take great care of your mental health” (P11). One study^21^ found that health care teams providing care to patients with COVID-19 had high levels of anxiety and stress.

Avoiding a purely negative outlook, ie, looking for opportunities in the midst of difficulties, was also reported as a way to alleviate the anguish of the situation. “Isolation was peaceful. For me, it was not serious at all. Staying at home, in fact, for those who never stay at home, was really nice” (P5). “Isolation wasn’t bad for me because I was with my family. So, the biggest fear was that something would happen to the children” (P7). “These are moments with the family” (P13). “The most important thing in our lives is God and family, because if it weren’t for my family, if I had been in isolation [alone], I think I would have died” (P6). There was a clear association between being with family and mental health.

### FAITH

Thinking about things that “brought them hope”^19^ helped the participants deal with the dark adversities of frontline work. “The big issue is dealing with emotion, the most difficult part of adapting to people. As a way of coping, we have faith… we have really trusted in God, we have said our prayers…” (P4). “It was faith, the family’s love” (P6).

“Mental health is linked to how you organize what is around you internally… trying to be positive. How they spoke of the importance of faith! Believing that better days will come” (P8). “Most people are letting their feelings be shaken. Many are extremely stressed…I have advised [my colleagues to seek] more balanced emotional health” (P4). “[C]olleagues [were] calling me sometimes, wanting to listen; I welcomed everyone, even though they weren’t doing so well...everyone around me caught COVID-19, work colleagues, family. I always tried to maintain and convey positivity that things will work out” (P8). “I used my faith in God as a strategy, faith that there is someone who can take care of us and can give us the ability to get through this difficult time” (P5). “[B]elieve that God will take care [of you], because you find yourself in a situation where you have no control” (P7).

A review^22^ of 19 articles revealed that religious coping and trust in God are strongly correlated with lower stress levels. They are also related to well-being and predict faster resolution of mental health problems. Various participant statements indicated that spirituality was an important factor in better coping with the pandemic.

### LESSONS LEARNED

Day by day, new experiences brought greater knowledge and better insight about how to deal with the difficulties of the pandemic. “We have already learned a lot about the disease; our techniques have advanced since the pandemic started” (P5). “I hope it doesn’t happen again, but if it does, we will be better prepared to act and guide patients through all procedures. But what I think could have been different is political preparation through more accurate public measures” (P5). “We really liked the staff’s testimonials. We have learned during this pandemic that we must value each other; at work we depend on each other… the pandemic has made us all equal” (P4).

Finally, participating in the research focus group also helped the participants, since listening to each other and sharing, in addition to bringing meaning to the worker’s experience, also led to the understanding that, although they were doing their part, they needed each other to get through the pandemic.

## CONCLUSIONS

By investigating the experiences of health workers, this study brought their main concerns to light, which included contracting the virus and, consequently, infecting their family members, as well as taking sick leave and overloading their colleagues. They were also worried about losing co-workers or their family members to the pandemic.

Mental health care, routines, faith, and learning lessons stood out as coping mechanisms on an individual level, while prevention measures, such as the use of masks, alcohol gel, social distancing, and social isolation, stood out on a collective level.

Promoting mental health among health care workers still faces a number of challenges, one of which is creating opportunities to listen, which produces meaning, empathy, and recognition. Health education must also be strengthened, including preventive measures for the population and worker support networks. We conclude, therefore, that these worker’s experiences both enrich our knowledge and indicate that further exploration is needed.
